# Two new species of the genus *Abrus* Dai & Zhang, 2002 (Hemiptera, Cicadellidae, Deltocephalinae) from China

**DOI:** 10.3897/zookeys.419.7481

**Published:** 2014-06-24

**Authors:** Jichun Xing, Zizhong Li

**Affiliations:** 1 Institute of Entomology, Guizhou University; Guizhou Key Laboratory for Plant Pest Management of Mountainous Region, Guizhou University, Guiyang, Guizhou Province, 550025, China.

**Keywords:** Homoptera, leafhopper, morphology, taxonomy, distribution

## Abstract

Two new species of leafhoppers: *Abrus damingshanensis* **sp. n.** (from Guangxi) and *A. expansivus* **sp. n.** (from Guizhou) are described and illustrated from China. A map showing the geographic distribution of the two new species is given. Taxonomic notes on species of the genus *Abrus* is also provided.

## Introduction

The genus *Abrus* belonging to the tribe Athysanini of subfamily Deltocephalinae, was established by Dai and Zhang (2002) with six species: *Abrus hengshanensis*, *Abrus brevis*, *Abrus huangi*, *Abrus wuyiensis*, *Abrus bifurcatus* and *Abrus coneus* from China and with *Abrus hengshanensis* as its type species. It belongs to the tribe Athysanini of subfamily Deltocephalinae (Hemiptera: Cicadellidae). Later, Li and Wang (2006) described two new species: *Abrus concavelus* and *Abrus leigongshanensis*. Dai and Zhang (2008) reviewed this genus and added a new species *Abrus breviolus*. Recently, Li (in Li et al. 2011) described two new species: *Abrus biprocessus* and *Abrus graciaedeagus*, and recorded *Abrus brevis*, *Abrus coneus* and *Abrus leigongshanensis* feeding on bamboo. Chen, Yang and Li (2012) described four new species, namely, *Abrus anlongensis*, *Abrus bambusanus*, *Abrus daozhenensis* and *Abrus yunshanensis*. Yang and Chen (2013) described two new species: *Abrus xishuiensis* and *Abrus langshanensis*, and provided a key to 13 known species. Morevoer, *Abrus brevis*, *Abrus coneus*, *Abrus leigongshanensis*, *Abrus anlongensis*, *Abrus bambusanus*, *Abrus daozhenensis*, *Abrus yunshanensis*, *Abrus xishuiensis* and *Abrus langshanensis* were recorded that they feed on bamboo (Li et al. 2011; Chen et al. 2012; Yang and Chen 2013). So far, 17 species of this genus were known from China, of them, all species are distributed in the Oriental Region (China: Guizhou, Sichuan, Hunan, Hubei, Guangxi, Guangdong, Fujian and Zhejiang), and only *Abrus coneus* is also distributed in the Palaearctic Region (China: Gansu).

This genus is distinguished by its crown with two pairs of similar black spots on anterior margin, clypellus expanded apically, male pygophore with a long membranous process from its inner apex, and aedeagus with a well-developed basal projection dorsally (except *Abrus breviolus* and *Abrus langshanensis*).

In the present paper, two new species: *Abrus damingshanensis* sp. n. and *Abrus expansivus* sp. n. are described and illustrated from China (Oriental Region, Fig. 23). The type specimens of the new species are deposited in the Institute of Entomology, Guizhou University, Guiyang, China (GUGC).

## Material and methods

Terminology of morphological and genital characters follow Dai and Zhang (2008). Male specimens were used for the description and illustration. External morphology was observed under a stereoscopic microscope and characters were measured with an ocular micrometer. Color pictures for adult habitus were obtained by KEYENCE VHX-1000 system. The genital segments of the examined specimens were macerated in 10% NaOH and drawn from preparations in glycerin jelly using a Leica MZ 12.5 stereomicroscope. Illustrations were scanned with Canon CanoScan LiDE 200 and imported into Adobe Photoshop CS8 for labeling and plate composition.

## Descriptions of species

### Abrus damingshanensis

Taxon classificationAnimaliaHemipteraCicadellidae

Xing & Li sp. n.

http://zoobank.org/211F15BE-9FD5-4A84-9D74-E3A16BA498DD

Figs 1–11

#### Description.

Yellowish brown species. Crown with two pairs of similar blackish brown spots on anterior margin. Eyes brown. Ocelli pale yellow. Pronotum with yellowish-brown stripe on anterior part. Inner and central anteapical cells at apex, third and fourth apical cells at base each with a dark brown spot. Face brown, frontoclypeus yellowish brown. Forewings yellowish. Legs marked with brown.

Body elongate, robust. Head including eyes narrower than greatest width of pronotum. Vertex with fore margin produced roundly, median length shorter than width between eyes. Eyes fairly large. Ocelli on anterior margin, separated from corresponding eye by approximately their own diameter. Frontoclypeus distinctly longer than wide, anteclypeus expanded apically. Antennae arising near lower corner of eye. Pronotum with anterior margin strongly and roundly produced, posterior margin slightly concave. Scutellum triangular, slightly shorter than pronotum, with transverse suture curved and depressed. Forewing with 3 subapical and 4 apical cells, 4 times as long as wide, appendix wide. Hind wings with three apical cells and two anteapical cells. Profemur with 2 dorsoapical setae. Hind femur apical setal formula 2+2+1. Hind tibia flattened and nearly straight, with PD setae very long. Metabasitarsomere with three platellae and two setae on apical transverse row.

*Male genitalia*. Male pygofer side longer than high, with many macrosetae posteriorly and some at midventral margin; posterior margin lobe alongate and with a long membranous process at inner apex (Fig. 5). Valve subtriangular with anterior margin concaved and posterior margin strongly produced medially (Fig. 6). Subgenital plate narrowing to rounded apex, outer margin rounded, with uniseriate row of macrosetae along lateral margin (Fig. 7). Aedeagus with well-developed basal projection on dorsal margin, tapered to acute apex, with pair of dorsal quadrilateral flange at midlength on dorsal margin; aedeagal shaft about half length of basal projection, expanded medially, apically branched in the caudal view, gonopore apical (Figs 8, 9). Connective Y–shaped, stem robust, arms well developed, articulated with the aedeagus (Fig. 10). Style long, broad at base, narrow at middle, apical margin expanded (Fig. 11).

**Figures 1–11.**
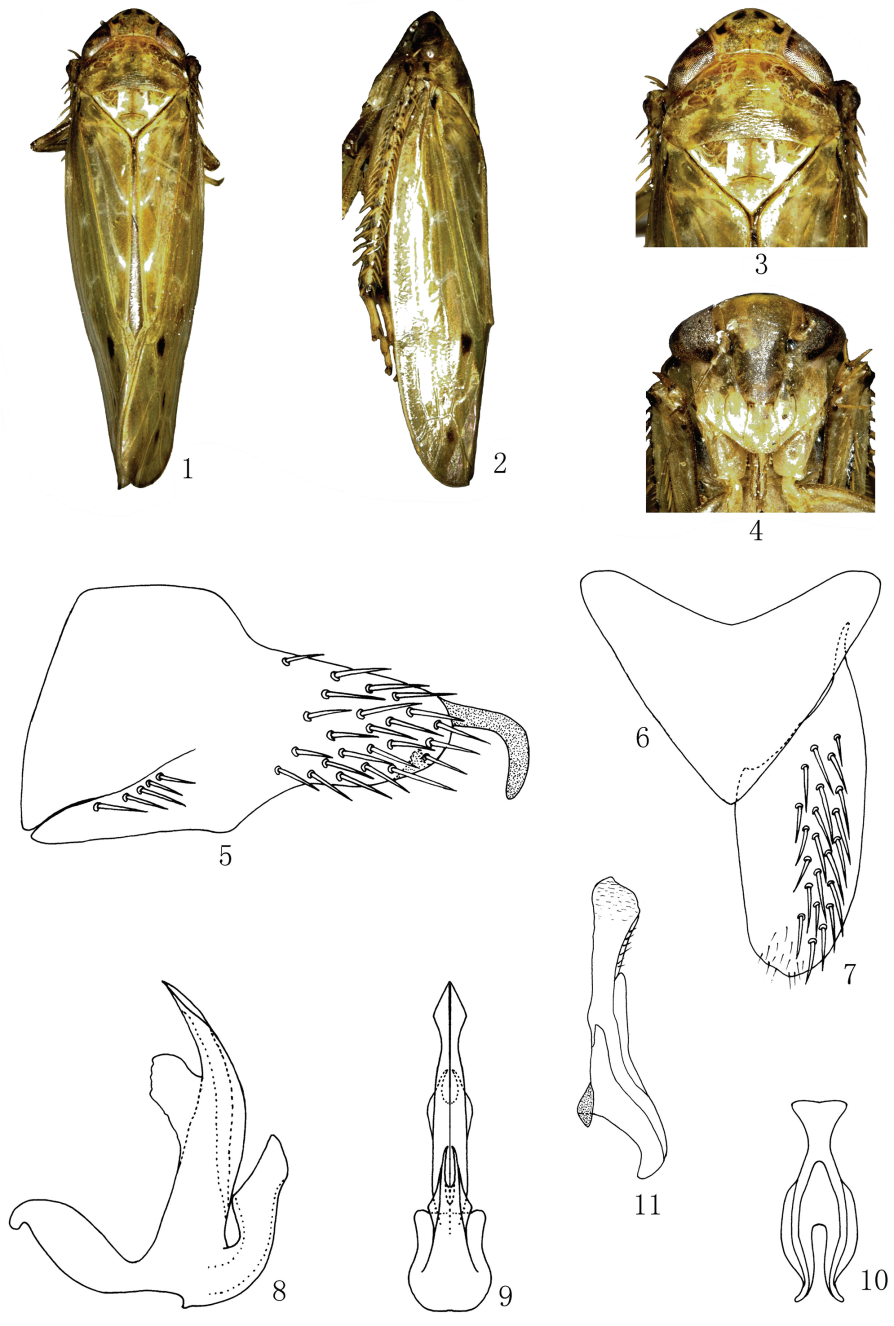
*Abrus damingshanensis* sp. n., **1** ♂, dorsal view **2** ♂, lateral view **3** ♂, head and thorax, dorsal view **4** ♂, face **5** Male pygofer side, lateral view **6** Valve, ventral view **7** Subgenital plate, ventral view **8** Aedeagus, lateral view **9** Aedeagus, caudal view **10** Connective, ventral view **11** Style, dorsal view.

#### Measurement.

Length (including tegmen): ♂, 9.1–9.2 mm.

#### Type material.

Holotype ♂, **China: Guangxi** Autonomous Region, Nanning City, Mt. Damingshan, 13 August 2011, coll. Zaihua Yang (GUGC); paratypes 2♂♂, same data as holotype (GUGC).

#### Diagnosis.

Thenew species is similar to *Abrus leigongshanensis* Li & Wang, 2006, but can be distinguished from the latter by the aedeagal shaft broad and short (about half length of basal projection); the basal projection tapered to acute apex, with pair of quadrilateral flange at midlength; and the apical process of style expanded.

#### Etymology.

This new species is named after the type locality, Damingshan, Guangxi Autonomous Region in China.

### Abrus expansivus

Taxon classificationAnimaliaHemipteraCicadellidae

Xing & Li sp. n.

http://zoobank.org/9C25F88C-D706-481F-B6B1-089C0EE2D4D8

Figs 12–22

#### Description.

External features resemble as *Abrus damingshanensis* sp. n., but color light yellow and body slightly small.

*Male genitalia*. Male pygofer side elongate with many macrosetae posteriorly and a few at midventral margin; posterior margin lobe elongate and with a long membranous process at inner apex (Fig. 16). Valve subtriangular with anterior margin concaved and posterior margin strongly produced medially (Fig. 17). Subgenital plate broad and short, outer margin rounded, with many macrosetae on lateral margin (Fig. 18). Aedeagus with broad and flat basal projection from dorsal margin, deeply concave at dorsal margin, the basal projection without processes; aedeagal shaft slightly shorter than basal projection, with a pair of slender apical processes and its length equal to aedeagal shaft, gonopore apical (Figs 19, 20). Connective Y–shaped, stem robust, arms well developed, articulated with the aedeagus (Fig. 21). Style long, broad at base, narrow at middle, apex slightly widening (Fig. 22).

**Figures 12–22.**
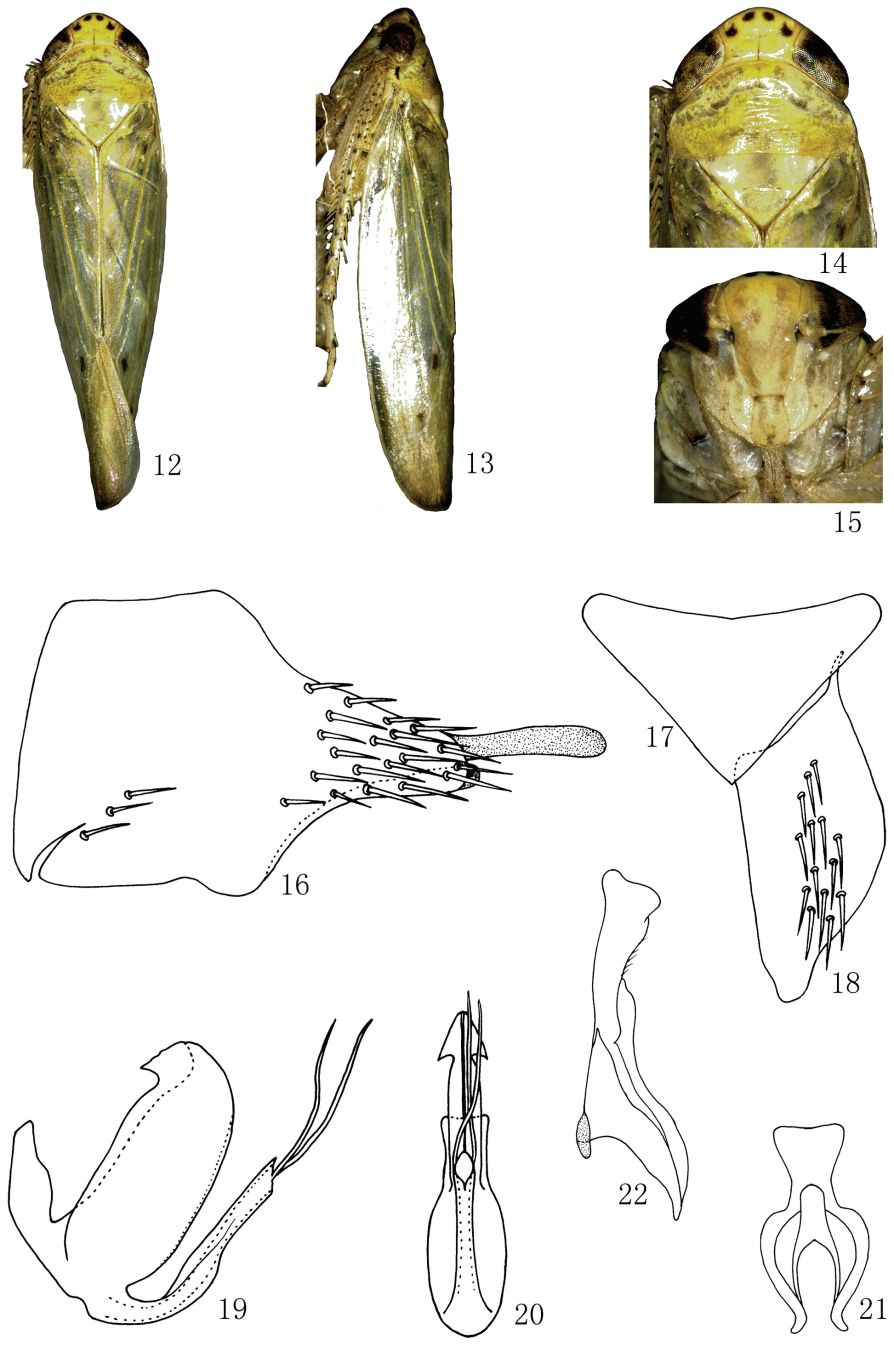
*Abrus expansivus* sp. n., **12** ♂, dorsal view **13** ♂, lateral view **14** ♂, head and thorax, dorsal view **15** ♂, face **16** Male pygofer side, lateral view **17** Valve, ventral view **18** Subgenital plate, ventral view **19** Aedeagus, lateral view **20** Aedeagus, caudal view **21** Connective, ventral view **22** Style, dorsal view.

#### Measurement.

Length (including tegmen): ♂, 8.1 mm.

#### Type material.

Holotype ♂, **China: Guizhou** Province, Dushan County, 16 July 2012, coll. Qiongzhang Song (GUGC).

#### Diagnosis.

This new species is very similar to *Abrus brevis* Dai & Zhang, 2002 in aedeagal shaft with a pair of long apical appendages, but can be distinguished from the latter by the aedeagal shaft longer than half length of basal projection; aedeagal shaft with apical processes located medially in lateral view and its length equal to aedeagal shaft; the apical process of style wide and flat; and the subgenital plate narrow apically.

#### Etymology.

The new species name is derived from the Latin word “*expansivus*”, referring to the apical process of style wide and expand.

## Taxonomic notes on *Abrus* species

Species of *Abrus* are all very similar in coloration and difficult to distinguish externally, but the structure of aedeagus are markedly different. This genus now contains 19 species, them can be divided into 3 types based on the structure of aedeagus: 1) basal projection of aedeagus very small or absent (*Abrus breviolus* and *Abrus langshanensis*); 2) aedeagal shaft obviously shorter than basal projection (*Abrus brevis*, *Abrus leigongshanensis*, *Abrus damingshanensis* sp. n. and *Abrus expansivus* sp. n.); 3) aedeagal shaft as long as or longer than basal projection (other 13 species).

Above mentioned the second type structure of aedeagus, of them, two species (*Abrus brevis* and *Abrus damingshanensis* sp. n.) with aedeagal shaft about half length of basal projection, two species (*Abrus brevis* and *Abrus expansivus* sp. n.) with aedeagal shaft apically have a pair of slender processes, and two species (*Abrus damingshanensis* sp. n. and *Abrus expansivus* sp. n.) with the apical process of style expand.

**Figure 23.**
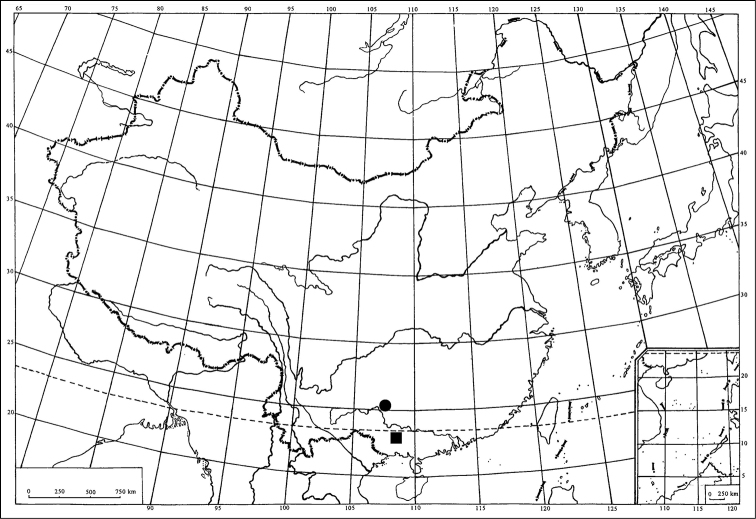
Geographic distribution of two new *Abrus* species in China: *Abrus damingshanensis* sp. n. (■); *Abrus expansivus* sp. n. (●).

## Supplementary Material

XML Treatment for Abrus damingshanensis

XML Treatment for Abrus expansivus
